# Probing an Interfacial Surface in the Cyanide Dihydratase from *Bacillus pumilus*, A Spiral Forming Nitrilase

**DOI:** 10.3389/fmicb.2015.01479

**Published:** 2016-01-05

**Authors:** Jason M. Park, Andani Mulelu, B. Trevor Sewell, Michael J. Benedik

**Affiliations:** ^1^Department of Biology, Texas A&M University, College StationTX, USA; ^2^Structural Biology Research Unit, Department of Integrative Biomedical Sciences, Institute for Infectious Diseases and Molecular Medicine, University of Cape TownCape Town, South Africa

**Keywords:** nitrilase, cyanide dihydratase, cyanide, bioremediation, oligomerization surface, quaternary structure

## Abstract

Nitrilases are of significant interest both due to their potential for industrial production of valuable products as well as degradation of hazardous nitrile-containing wastes. All known functional members of the nitrilase superfamily have an underlying dimer structure. The true nitrilases expand upon this basic dimer and form large spiral or helical homo-oligomers. The formation of this larger structure is linked to both the activity and substrate specificity of these nitrilases. The sequences of the spiral nitrilases differ from the non-spiral forming homologs by the presence of two insertion regions. Homology modeling suggests that these regions are responsible for associating the nitrilase dimers into the oligomer. Here we used cysteine scanning across these two regions, in the spiral forming nitrilase cyanide dihydratase from *Bacillus pumilus* (CynD), to identify residues altering the oligomeric state or activity of the nitrilase. Several mutations were found to cause changes to the size of the oligomer as well as reduction in activity. Additionally one mutation, R67C, caused a partial defect in oligomerization with the accumulation of smaller oligomer variants. These results support the hypothesis that these insertion regions contribute to the unique quaternary structure of the spiral microbial nitrilases.

## Introduction

Nitrilase enzymes from the large nitrilase-superfamily are of significant industrial interest due to their ability to process nitrile compounds into valuable acid products such as nicotinic acid, acrylic acid, and glycolic acid (Singh et al., 2006; Gong et al., 2012). They also offer economic and environmentally friendly alternatives to current hazardous and costly methods of detoxifying nitrile wastes (Korte and Coulston, 1995; Gong et al., 2012). One of the most prevalent and certainly the most toxic nitrile waste is cyanide, which has extensive uses in industry from polymer synthesis to mining and electroplating (Korte and Coulston, 1995; Akcil, 2003). Nitrilases such as cyanide dihydratase (CynD) provide an attractive option for degrading the high volumes of cyanide wastes produced by these industries (Meyers et al., 1993; Watanabe et al., 1998).

In order to exploit further application of these nitrilases, the enzymes often need to be engineered to tolerate industrial conditions or recognize specific substrates. While random mutagenesis and high throughput screening have revealed useful mutants (Schreiner et al., 2010; Abou Nader, 2012; Wang et al., 2012), our ability to make rational changes is impeded by the absence of structural information of these enzymes. Three dimensional, negative stain electron microscopy (3D EM) has shown that many active nitrilases form large spiral shaped oligomers (Jandhyala et al., 2003; Sewell et al., 2003; Thuku et al., 2007; Woodward et al., 2008; Dent et al., 2009; Williamson et al., 2010).

The spiral structure has also been linked to the activation and substrate specificity of nitrilases. This is highlighted by the example of the *Rhodococcus rhodochrous* J1 nitrilase which was purified as an inactive dimer. These dimers formed decamers when incubated with the substrate benzonitrile. Once activated by oligomerization, the nitrilase is able to recognize additional substrates that were themselves unable to activate the enzyme (Nagasawa et al., 2000).

Cyanide is the smallest nitrile and is a substrate for two different types of nitrilases. The cyanide dihydratases convert cyanide to formate and ammonia, whereas the cyanide hydratases generate formamide which is often subsequently converted to formate and ammonia by the action of independent amidases. Typically cyanide dihydratases (CynD) are found in bacteria and the cyanide hydratases (CHT) are found in fungi. Both are found as the typical oligomeric spirals (Jandhyala et al., 2005; Thuku et al., 2009).

All nitrilase superfamily members share a common E-K-C-E active site. Subtle positioning of these residues, and access of water molecules, creates the diverse range of carbon nitrogen chemistry within this family. Spiral formation in nitrilases is needed to fully form the active site (Lundgren et al., 2008).

The CynD protein from *Bacillus pumilus* forms a self-terminating, spiral 18-mer at neutral pH. CynD does not require substrate to oligomerize and is found only as oligomers. However, the spiral length can be altered. When the pH is lowered to 5.4, longer helices of variable length are formed. These are readily measured to have a helical rise (Δz) of 1.62 nm and a left handed helical twist (ΔΦ) of 77° (Jandhyala et al., 2003). The elongation is also associated with a slight increase in CynD activity thought to stem from the activation of formerly terminal subunits which now interact across the elongation interface (Jandhyala et al., 2005). This explanation is consistent with the observation that terminal monomers in the oligomer of the nitrilase-like beta-alanine synthase (βaS) from *Drosophila melanogaster* (Lundgren et al., 2008) can be seen as having defective active sites.

The quaternary structures of the majority of the crystallized members of the nitrilase superfamily (Nakai et al., 2000; Pace et al., 2000; Wang et al., 2001; Kumaran et al., 2003; Kimani et al., 2007; Lundgren et al., 2008), most of which exhibit amidase or carbamylase activity, do not resemble the spirals that are seen by electron microscopy. In the case of several enzymes that exhibit nitrilase activity, however, an αββα fold is highly conserved as is the dimer forming interface. The monomers associate across the A-surface to form the αββα-αββα dimer (Nakai et al., 2000; Pace et al., 2000; Wang et al., 2001; Kumaran et al., 2003; Kimani et al., 2007; Lundgren et al., 2008). To understand how these dimers may interact to form the spiral structure, the dimers were fitted into the reconstructions obtained by 3D EM (Sewell et al., 2003, 2005; Thuku et al., 2007; Woodward et al., 2008; Dent et al., 2009; Williamson et al., 2010). The docked dimers enable pseudo-atomic models of the elongated fibers to be obtained, and these models in turn enable the identification of those amino acids that may play a role in the interface.

**FIGURE 1:**
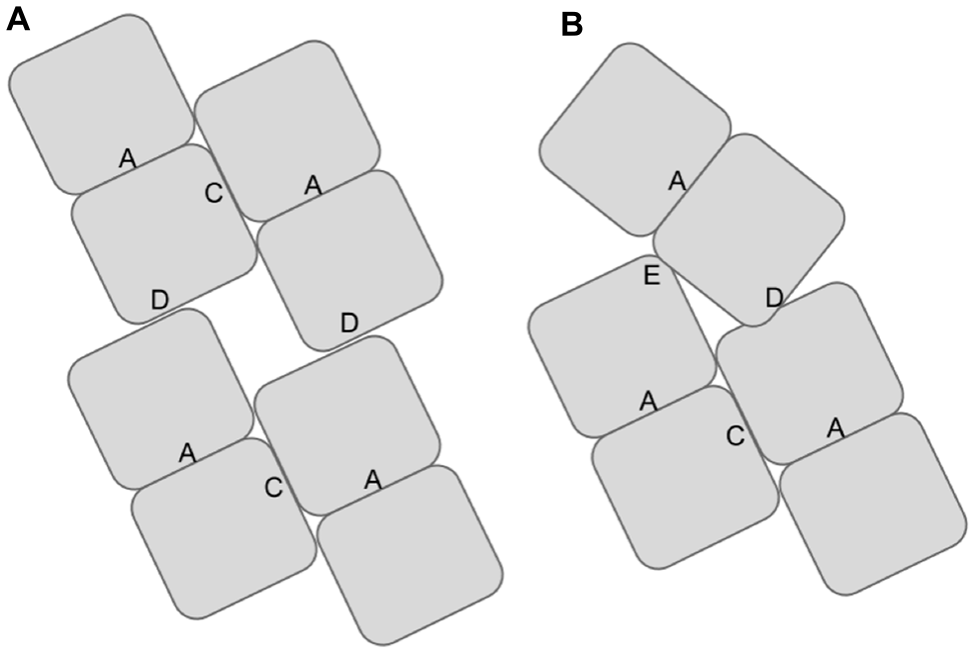
**Diagram showing the locations of the associating surfaces within spirals formed by members to the nitrilase superfamily.** Monomers are represented by rectangles. These monomers associate to form dimers at the A-surface in all superfamily members. The dimers associate to form helical assemblies by associating across the C-surface. Two dimers are shown per turn in **(A)**. In some superfamily members such as CynD interactions occur across the groove leading to the formation of the D-surface between adjacent turns of the spiral. In CynD, alternative interactions across the groove leading to the formation of the E-surface **(B)** cause to the termination of the spirals after a fixed number of dimers.

The reconstruction of the spirals and helices show evidence of a large interacting surface that is approximately perpendicular to the dimerization interface. This interface between dimers plays a primary role in the elongation of the spiral and has been referred to as the C-surface (Sewell et al., 2003). Thus the interacting amino acids on adjacent molecules are related by twofold axes. This C-surface interaction is one of two classes of interactions of the dimers seen in the fully elongated spirals. Other interactions form once the spiral completes a full turn. The details of these interactions appear to vary depending on the nitrilase. They include the cross-spiral D and F-surfaces, which may play some role in stabilizing the long helices and the E-surface, which appears to stop further spiral extension (**Figure 1**; Sewell et al., 2005; Thuku et al., 2007; Woodward et al., 2008; Dent et al., 2009).

**FIGURE 2:**
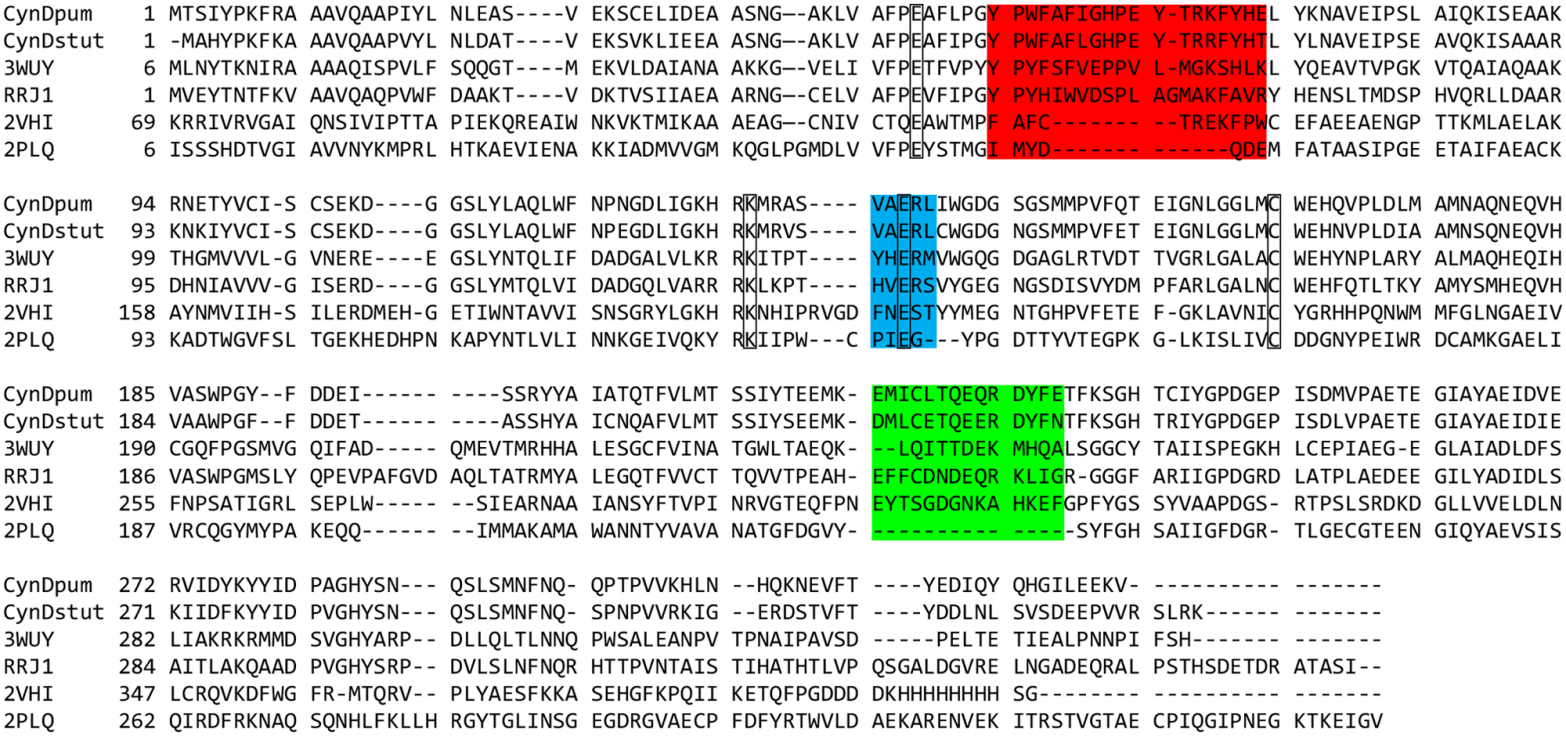
**Multiple sequence alignment of: cyanide dihydratase from *Bacillus pumilus* C1 and *Pseudomonas stutzeri* (CynDpum, CynDstut) (Meyers et al., 1993; Jandhyala et al., 2003; Sewell et al., 2003), oxy-nitrilase from *Synechocystis* PCC6803 (3WUY) (Zhang et al., 2014), nitrilase from *R. rhodochrous* J1 (RRJ1) (Thuku et al., 2007), C-shaped β-alanine-synthase (βaS) from *Drosophila melanogaster* (2VHI) (Lundgren et al., 2008), and non spiral-forming crystallized nitrilase superfamily member 2PLQ (Nakai et al., 2000; Pace et al., 2000; Wang et al., 2001; Kumaran et al., 2003; Kimani et al., 2007).** Sequence insertion regions in the spiral-forming nitrilases proposed to participate in the C-surface interaction leading to spiral formation are highlighted. Region 1 is highlighted in red and region 2 is highlighted in green. The putative catalytic residues are outlined. The sequence highlighted in blue also forms part of the interface and contributes a glutamate to the active site. The multiple sequence alignment was constructed with ClustalW2 (Larkin et al., 2007; Goujon et al., 2010). Alignment was edited and exported using Jalview software (Waterhouse et al., 2009).

The amino acids of the spiral forming nitrilases that comprise the putative C-surface have been identified by aligning the sequences of these enzymes to those of the members of the superfamily for which the crystal structures have been determined. Homology modeling locates two insertions in the spiral forming nitrilases relative to the non spiral-forming members of the superfamily comprising residues 55-72 and 222-235 in CynD, as participating in the formation of the C-surface (**Figure 2**; Thuku et al., 2009). In a prior study investigating the oligomeric surfaces in cyanide dihydratase from *B. pumilus* (CynD), the C-surface region 2 (residues 222-235) was deleted. This lead to complete loss of activity but the effect on oligomerization was not examined (Sewell et al., 2005). The other insertion, C-surface region 1 (55-72), has not to-date been analyzed.

The crystal structures of the *D. melanogaster* beta-alanine synthase (βaS; Lundgren et al., 2008) and the oxy-nitrilase from *Synechocystis* sp. strain PCC6803 (Zhang et al., 2014) suggest possible conformations for these insertions and confirm the role that they play in creating the spiral-forming interface. βaS forms C-shaped spiral oligomers and has one insertion (corresponding to region 2) relative to the other crystallized nitrilase-superfamily members. The insertion forms a loop that lies at an intermolecular interface corresponding to the C-surface identified in the 3D EM reconstructions of extended spirals. The amino acids in the loop interact with the twofold related loop in the adjacent molecule. The other insertion in CynD, 55-72, is eight residues shorter in βaS than in CynD. However, both insertions in the *Synechocystis* oxy-nitrilase are of similar length to those in CynD, and their association in the crystal leads to the formation of an extended helix with sixfold symmetry. The conformation of the loop that corresponds to residues 222-235 in CynD is completely different to that of the corresponding loop in βaS.

To test the involvement of these putative C-surface regions in spiral formation, we scanned the two regions in CynD for mutations affecting the oligomeric state or activity of the protein. Individual cysteine substitutions at each residue within these two putative C-surface regions were constructed to search for the possibility of disulfide bridge formation at one or more positions. Each of these mutants was characterized for effects on enzymatic activity and the oligomeric state as deduced from size exclusion gel chromatography.

## Materials and Methods

### Molecular Modeling

Models of CynD based on the co-ordinates of either the nitrilase-like beta-alanine synthase (βaS) from *Drosophila melanogaster* (PDB ID:2VHI), or the oxy-nitrilase from *Synechocystis* sp. strain PCC6803 (PDB ID:3WUY), were generated using the alignments shown in **Figure 2** using Modeller (Sali and Blundell, 1993) running within UCSF Chimera (Pettersen et al., 2004). The molecular graphic images were generated using UCSF Chimera.

### Media and Reagents

Luria broth and plates were used for the growth of all bacterial strains, supplemented as needed with 25 μg/ml chloramphenicol or 25 μg/ml kanamycin. Phusion High Fidelity DNA polymerase master mix and restriction enzymes were purchased from New England Biolabs (NEB; Ipswich, MA). B-PER^®^ extraction reagents, HisPur^TM^ Cobalt Spin Columns, and Zeba Desalting Columns were obtained from Thermo-Scientific (Pierce Biotechnology; Rockford, IL, USA).

### Bacterial Strains Plasmids

*Escherichia coli* strain MB3436 (Δ*endA thiA hsdR17 supE44 lacI^q^ZΔm15*) was used for cloning and mutant construction. Plasmids were transformed into *E. coli* BL21(DE3) pLysS for expression. All substitution mutants were constructed in pMB4407, which is pET28a carrying *cynD* as a NdeI-XhoI insertion (Abou Nader, 2012).

### Scanning Mutagenesis

Mutants were constructed by site directed mutagenesis following QuickChange^®^ protocol (Stratagene, La Jolla, CA, USA) using mutagenic primers (**Table 1**) with Phusion DNA polymerase and confirmed by sequencing.

**Table 1:** Primers used in the construction of the substitution mutations.

C-surface regions site directed mutagenesis primers	
Pum Y54C F	GAA GCA TTT TTA CCT GGT TGC CCT TGG TTT GCT TTT ATT G
Pum Y54C R	CAA TAA AAG CAA ACC AAG GGC AAC CAG GTA AAA ATG CTT C
Pum P55C F	GCA TTT TTA CCT GGT TAT TGC TGG TTT GCT TTT ATT GG
Pum P55C R	CCA ATA AAA GCA AAC CAG CAA TAA CCA GGT AAA AAT GC
Pum W56C F	CCT GGT TAT CCT TGC TTT GCT TTT ATT GG
Pum W56C R	CCA ATA AAA GCA AAG CAA GGA TAA CCA GG
Pum F57C F	CTG GTT ATC CTT GGT GTG CTT TTA TTG GTC
Pum F57C R	GAC CAA TAA AAG CAC ACC AAG GAT AAC CAG
Pum A58C F	GGT TAT CCT TGG TTT TGT TTT ATT GGT CAT CC
Pum A58C R	GGA TGA CCA ATA AAA CAA AAC CAA GGA TAA CC
Pum F59C F	CCT TGG TTT GCT TGT ATT GGT CAT CC
Pum F59C R	GGA TGA CCA ATA CAA GCA AAC CAA GG
Pum I60C F	GTT ATC CTT GGT TTG CTT TTT GTG GTC ATC CAG AAT ATA CG
Pum I60C R	CGT ATA TTC TGG ATG ACC ACA AAA AGC AAA CCA AGG ATA AC
Pum G61C F	CCT TGG TTT GCT TTT ATT TGT CAT CCA GAA TAT ACG
Pum G61C R	CGT ATA TTC TGG ATG ACA AAT AAA AGC AAA CCA AGG
Pum H62C F	GTT TGC TTT TAT TGG TTG TCC AGA ATA TAC GAG
Pum H62C R	CTC GTA TAT TCT GGA CAA CCA ATA AAA GCA AAC
Pum P63C F	GTT TGC TTT TAT TGG TCA TTG CGA ATA TAC GAG AAA GTT C
Pum P63C R	GAA CTT TCT CGT ATA TTC GCA ATG ACC AAT AAA AGC AAA C
Pum E64C F	GCT TTT ATT GGT CAT CCA TGT TAT ACG AGA AAG TTC TAT C
Pum E64C R	GAT AGA ACT TTC TCG TAT AAC ATG GAT GAC CAA TAA AAG C
Pum Y65C F	GGT CAT CCA GAA TGT ACG AGA AAG TTC
Pum Y65C R	GAA CTT TCT CGT ACA TTC TGG ATG ACC
Pum T66C F	GGT CAT CCA GAA TAT TGC AGA AAG TTC TAT CAT G
Pum T66C R	CAT GAT AGA ACT TTC TGC AAT ATT CTG GAT GAC C
Pum R67C F	GTC ATC CAG AAT ATA CGT GCA AGT TCT ATC ATG
Pum R67C R	CAT GAT AGA ACT TGC ACG TAT ATT CTG GAT GAC
Pum K68C F	GTC ATC CAG AAT ATA CGA GAT GCT TCT ATC ATG A
Pum K68C R	TCA TGA TAG AAG CAT CTC GTA TAT TCT GGA TGA C
Pum F69C F	ATC CAG AAT ATA CGA GAA AGT GCT ATC ATG AAT TAT
Pum F69C R	ATA ATT CAT GAT AGC ACT TTC TCG TAT ATT CTG GAT
Pum Y70C F	CAG AAT ATA CGA GAA AGT TCT GCC ATG AAT TAT ATA AAA ATG C
Pum Y70C R	GCA TTT TTA TAT AAT TCA TGG CAG AAC TTT CTC GTA TAT TCT G
Pum H71C F	TAT ACG AGA AAG TTC TAT TGC GAA TTA TAT AAA AAT GCC G
Pum H71C R	CGG CAT TTT TAT ATA ATT CGC AAT AGA ACT TTC TCG TAT A
Pum E72C F	CGA GAA AGT TCT ATC ATT GCT TAT ATA AAA ATG CCG
Pum E72C R	CGG CAT TTT TAT ATA AGC AAT GAT AGA ACT TTC TCG
Pum E222C F	CGG AAG AAA TGA AAT GCA TGA TTT GTT TAA CG
Pum E222C R	CGT TAA ACA AAT CAT GCA TTT CAT TTC TTC CG
Pum M223C F	GAA ATG AAA GAG TGC ATT TGT TTA ACG CAG
Pum M223C R	CTG CGT TAA ACA AAT GCA CTC TTT CAT TTC
Pum I224C F	GAA ATG AAA GAG ATG TGC TGT TTA ACG CAG
Pum I224C R	CTG CGT TAA ACA GCA CAT CTC TTT CAT TTC
Pum C225A F	GAA AGA GAT GAT TGC TTT AAC GCA GGA G
Pum C225A R	CTC CTG CGT TAA AGC AAT CAT CTC TTT C
Pum L226C F	GAG ATG ATT TGT TGC ACG CAG GAG CAA AG
Pum L226C R	CTT TGC TCC TGC GTG CAA CAA ATC ATC TC
Pum K221C F	TAC GGA AGA AAT GTG CGA GAT GAT TTG
Pum K221C R	AAC AAA TCA TCT CGC ACA TTT CTT CCG
Pum T227C F	GAT GAT TTG TTT ATG CCA GGA GCA AAG AG
Pum T227C R	CTC TTT GCT CCT GGC ATA AAC AAA TCA TC
Pum Q228C F	GAT GAT TTG TTT AAC GTG TGA GCA AAG AGA TTA C
Pum Q228C R	GTA ATC TCT TTG CTC ACA CGT TAA ACA AAT CAT C
Pum E229C F	GAT TTG TTT AAC GCA GTG CCA AAG AGA TTA CTT TG
Pum E229C R	CAA AGT AAT CTC TTT GGC ACT GCG TTA AAC AAA TC
Pum Q230C F	GTT TAA CGC AGG AGT GCA GAG ATT ACT TTG
Pum Q230C R	CAA AGT AAT CTC TGC ACT CCT GCG TTA AAC
Pum R231C F	AAC GCA GGA GCA ATG CGA TTA CTT TGA AAC
Pum R231C R	GTT TCA AAG TAA TCG CAT TGC TCC TGC GTT
Pum D232C F	CGC AGG AGC AAA GAT GTT ACT TTG AAA C
Pum D232C R	GTT TCA AAG TAA CAT CTT TGC TCC TGC G
Pum Y233C F	CAG GAG CAA AGA GAT TGT TTT GAA ACA TTT AAG
Pum Y233C R	CTT AAA TGT TTC AAA ACA ATC TCT TTG CTC CTG
Pum F234C F	GCA AAG AGA TTA CTG CGA AAC ATT TAA GAG C
Pum F234C R	GCT CTT AAA TGT TTC GCA GTA ATC TCT TTG C
Pum E235C F	CAA AGA GAT TAC TTT TGC ACA TTT AAG AGC GG
Pum E235C R	CCG CTC TTA AAT GTG CAA AAG TAA TCT CTT TG


*Primers designed Primers designed using PrimerX (http://www.bioinformatics.org/primerx/).*

### Protein Expression and Purification

Protein was produced from *E. coli* BL21(DE3) pLysS transformed with pMB4407 or its derivatives. Cells were grown at 37°C to an OD600 between 0.4 and 0.6, and induced by adding IPTG to 1 mM and transferred to 30°C for 2-3 hour. Enzyme activities in whole cells were examined immediately after the end of induction.

Cells destined for lysate production and/or protein purification were pelleted at 5,000 g for 10 min and frozen at -20°C. Lysates were prepared using B-PER II Reagent^®^ with added lysozyme and DNase according to protocol (Pierce Biotechnology; Rockford, IL, USA). Lysates were diluted with an equal volume of wash buffer (50 mM sodium phosphate, 300 mM sodium chloride, 10 mM imidazole; pH 7.4). This was added to pre-equilibrated HisPur^TM^ Cobalt 0.2 ml resin bed Spin Columns (Pierce Biotechnology; Rockford, IL, USA) in two to three 600 μl applications. Each application of lysate was mixed end over end for 30 min on the sealed column. Columns were washed with 400 μl wash buffer three times, and the protein was eluted in 600 μl elution buffer (300 mM sodium chloride, 150 mM imidazole; pH7.4). Purified protein was exchanged into 0.1 M MOPS buffer ph7.7 using 2 ml Zeba^TM^ Spin Desalting Columns 7K MWCO (Pierce Biotechnology; Rockford, IL, USA) and stored on ice. Protein purity was assessed by the presence of only a single band on SDS-PAGE gels, and protein concentrations were measured by Bradford protein assay (BioRad Laboratories, Hercules, CA, USA). Sample stored longer than overnight were divided into 200 μl aliquots and frozen at -80°C.

### Chromatography

Purified protein samples in 0.1 M MOPS pH7.7 were separated at 0.5 ml/min on a Superdex^TM^ 200 10/300 GL column (Amersham Biosciences; Uppsala, Sweden) equilibrated with 0.1 M MOPS pH7.7 using a BioRad BioLogic DuoFlow^®^. Protein elution was monitored by absorbance at 220 and 280 nm using a BioRad BioLogic QuadTec^®^ UV-Vis detector. Dilute samples were first concentrated using Amicon Ultra-4 centrifugal filter devices 10,000 MWCO (Millipore; Billerica, MA). The column was calibrated using Sigma-Aldrich gel filtration marker kit containing; Carbonic Anhydrase, bovine erythrocytes (29,000 Da), Albumin, bovine serum (66,000 Da), Alchohol Dehydrogenase, yeast (150,000 Da), β-Amylase, sweet potato (200,000 Da), Apoferritin, horse spleen (443,000 Da), Thyroglobulin, bovine (669,000 Da).

### Purification for Electron Microscopy

Aliquots of the purified protein samples in 0.1M MOPS pH7.7 were exchanged into 50 mM Tris pH 7.7, 150 mM NaCl, buffer using spin columns (Microsep Pty Ltd., South Africa). The protein was then separated at 0.5ml/min on a Superdex^TM^ 200 10/300 GL column (Amersham Biosciences; Uppsala, Sweden) equilibrated with 50 mM Tris pH 7.7, 150 mM NaCl. Samples corresponding to the various elution peaks were collected and incubated overnight at 4°C before preparation of grids for electron microscopy.

### Electron Microscopy

The sample (3 μl) was incubated for 30 s on a glow-discharged carbon-coated copper grid, washed twice with deionized water, blotted with filter paper, and stained with three drops of 2% uranyl acetate, blotting between drops. Grids were examined using Tecnai T20 transmission electron microscopes operated at 200 kV, at a magnification of 69,000× and a nominal defocus of 3.0–5.0 μm. Images were recorded using a Gatan US1000 CCD camera.

### Intersubunit Disulfide Crosslinking

To examine disulfide bond formation across the C-surface of the cysteine mutant proteins, 3 μg of purified recombinant wild type or mutated CynD protein were analyzed using 10% SDS-PAGE gels under non-reducing conditions, and stained with coomassie blue to visualize protein bands. The appearance of higher molecular weight bands under non-reducing conditions indicated disulfide cross-linking among CynD monomers.

### Activity Assay

Activity was assayed using the picric acid method to detect cyanide as previously described (Wang et al., 2012). Purified protein was diluted to 50 μg/ml in 100mM MOPS pH7.7. From this dilution 10 μl was added to 80 μl of 100 mM MOPS pH7.7 in 96 well plates and allowed to incubate for 20 min at room temperature. To start the reaction, 20 μl of 25 mM KCN in 100 mM MOPS was added. The plate was covered with parafilm, which was pressed onto the wells to prevent evaporation of cyanide. The reaction was terminated at 20 min by adding 80 μl of alkaline picric acid (0.5% picric acid in 0.25 M sodium carbonate). To develop the color, the plate was incubated at 60°C for 20 min. Absorbance was measured at 520 nm in a Bio-Rad Benchmark Plus microplate spectrophotometer.

Activity from whole cells was measured similarly. 100 μl of culture was mixed with 100 μl of 6 mM KCN in 0.1 M MOPS pH 7.7 and the reaction was allowed to proceed at room temperature. The amount of remaining cyanide was detected by adding 100 μl of the reaction to 100 μl of alkaline picric acid (0.5% picric acid in 0.25 M sodium carbonate) in a 96 well plate and the absorbance was measured at 520 nm in a Bio-Rad Benchmark Plus microplate spectrophotometer.

## Results

### Modeling the C-Surface of CynD

**FIGURE 3:**
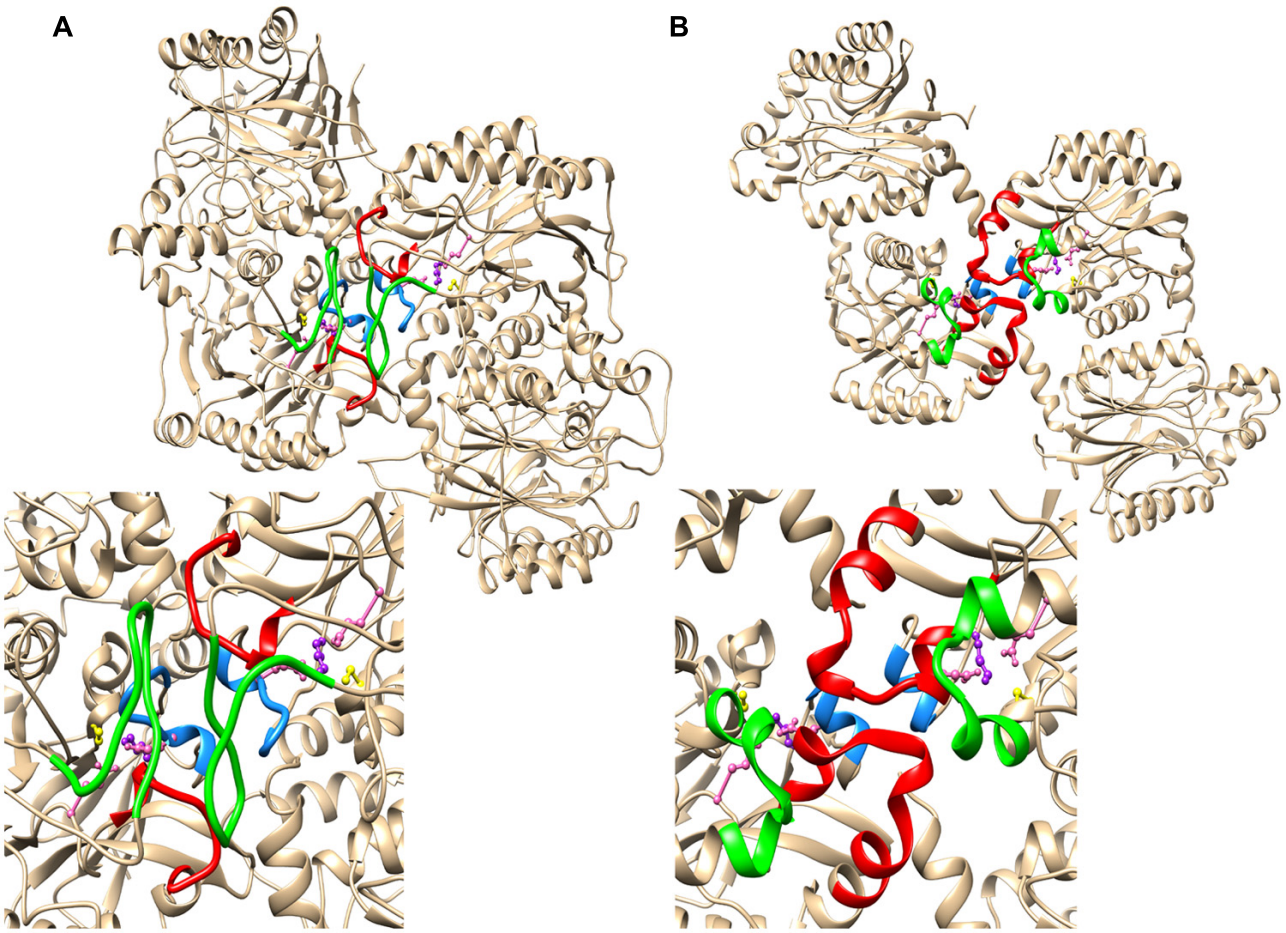
**The relationship between the region 1 and region 2 insertions and the C-surface based on crystal structures of two spiral forming members of the nitrilase superfamily **(A)** β-alanine synthase from *Drosophila melanogaster* (PDB id: 2VHI) and **(B)** the oxy-nitrilase from *Synechocystis* sp.** PCC6803 (PDB id: 3WUY). Region 1 is highlighted in red and region 2 is highlighed in green. In both cases there is a further contributor to the interface depicted in blue. This sequence contains a glutamate (depicted in pink) that is hydrogen bonded to the lysine of the putative catalytic triad comprising a cysteine (yellow), a glutamate (pink), and the lysine (purple).

The absence of a high resolution structure for CynD or a close analog necessitated modeling based on more distant homologs. The crystal structures of βaS (PDB id: 2VHI) and the oxy-nitrilase (PDB id: 3WUY) both show the twofold symmetry of the interactions across the C-surface, but in most respects the nature of the interactions and the conformation of the common insertion (corresponding to region 2) are different (**Figure 3**). In βaS, region 2 forms a loop between two strands of beta-sheet. The salt bridges formed between Glu298 and Lys306 are the main interactions leading to this association. The other component of the interface is the loop comprising residues 200 to 215 (corresponding to residues 131 to 146 in Cyn D). The spiral has a helical rise (Δz) of 1.41 nm and a left-handed helical twist (ΔΦ) of 83.1° which is slightly more tightly wound than CynD.

In oxy-nitrilase the dominant C surface interactions are between residues in region 1, which comprises two short helical segments linked by five amino acids that interact with the two-fold related segment in the adjacent molecule leading to the majority of the interfacial interactions. The interface is formed by hydrophobic interactions between Phe62 and Met71, Pro67 and Pro67, Val69 and Phe64, Leu70 and Met242 and there is a hydrogen bonding interaction between Tyr140 OH and the backbone carbonyl oxygen of Pro68. Region 2 of CynD differs from βaS in that it comprises two short α-helical segments linked by a bend containing four amino acids. The structure only contributes to the interface through Met242 as described above. Although the insertions are of similar length the spiral formed in the crystal is significantly different to that of CynD, having helical rise (Δz) of 2.68 nm and a left handed helical twist (ΔΦ) of 60.0°. Similar to βaS, the short α-helical region (140-144) that forms part of the loop following the active site lysine (K135) is located in the interface in such a way that an interaction between E142 and K135 is possible.

**FIGURE 4:**
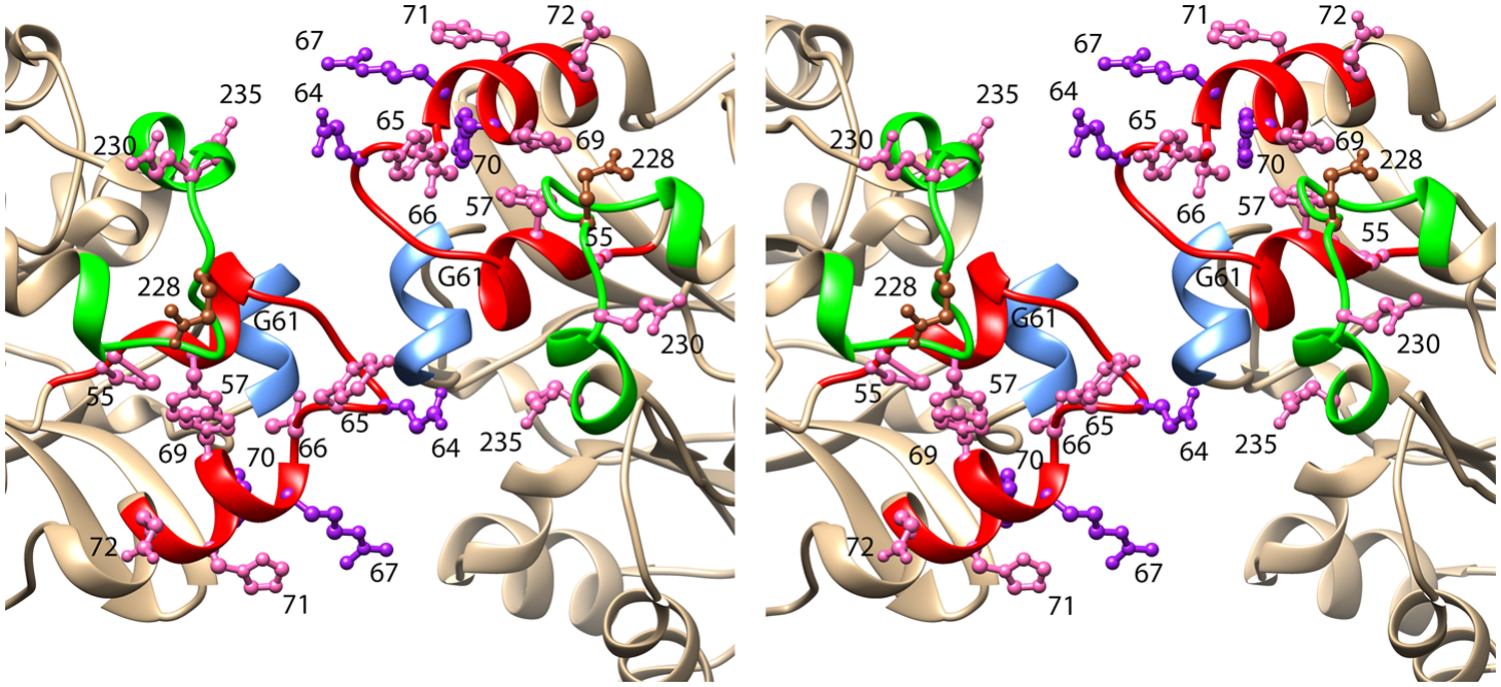
**Stereoview of the model of CynD based on the structure of the oxy-nitrilase from *Synechocystis* sp.** PCC6803 (PDB id: 3WUY). The ribbon depicting region 1 is colored red. This region includes three residues that when mutated to cysteine, caused CynD to lose activity: E64, R67, and Y70 (colored purple). It also included seven residues that when mutated to cysteine caused the activity to drop below 50% of the “wt” activity: P55, F57, Y65, T66, F69, H71, and E72 (colored pink). The ribbon depicting region 2 is colored green. It includes two residues that when mutated to cysteine caused the activity to drop below 50% of the “wt” activity: N230 and E235 (colored pink). Residue Q228, mutation of which caused aggregation of the short spirals without loss of activity is colored brown.

The secondary structure of CynD, predicted by PSIPRED (Jones, 1999) is very similar to that of the oxy-nitrilase and therefore the visualized residues in the structure PDB id: 3WUY were chosen as a template for CynD. The dimers thus modeled were arranged in a helix having Δz = 1.62 nm and a left handed helical twist ΔΦ = 77° with an outer diameter corresponding to that of the helices seen in the electron micrographs (11 nm). The model of the C surface thus obtained is depicted in **Figure 4.**

### Effect of C-Surface Mutations on Activity

**FIGURE 5:**
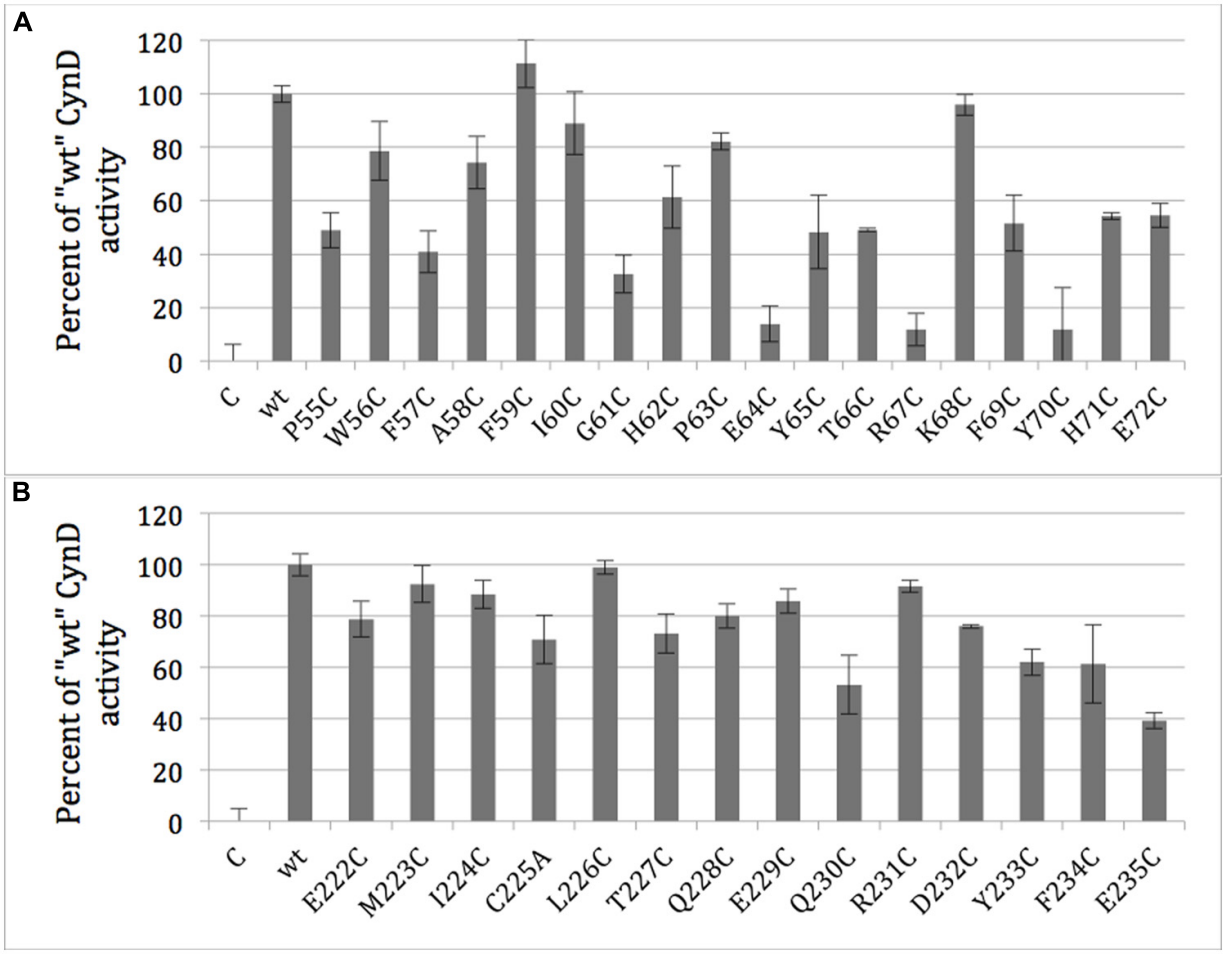
**Activity of purified CynD proteins relative to “wt” CynD and buffer only controls (C) measured by the picric acid CN assay.** Activity for each substitution mutant in the C-surface regions 1 (**A**, top) and 2 (**B**, bottom). Error bars show standard deviation from three samples.

Each residue within the two putative C-surface regions of CynD from *B. pumilus*, region 1 (P55-E72) and region 2 (E222-E235), was individually replaced with cysteine except the native cysteine (C225) was changed to alanine. The activity of each purified CynD variant protein was tested. CynD was found sensitive to cysteine substitution at several positions in C-surface region 1 (**Figure 5A**). Cysteine substitutions at P55, F57, G61, E64, Y65, T66, R67, F69, and Y70 reduced activity to ≤50% of wild type. Two of these variants, R67C and Y70C, had <10% initial activity. Additionally, prolonged reaction time did not result in further cyanide degradation. By contrast only two mutations in region 2, Q230C and E235C, reduced CynD activity to ≤50% (**Figure 5B**).

### Size Analysis

**FIGURE 6:**
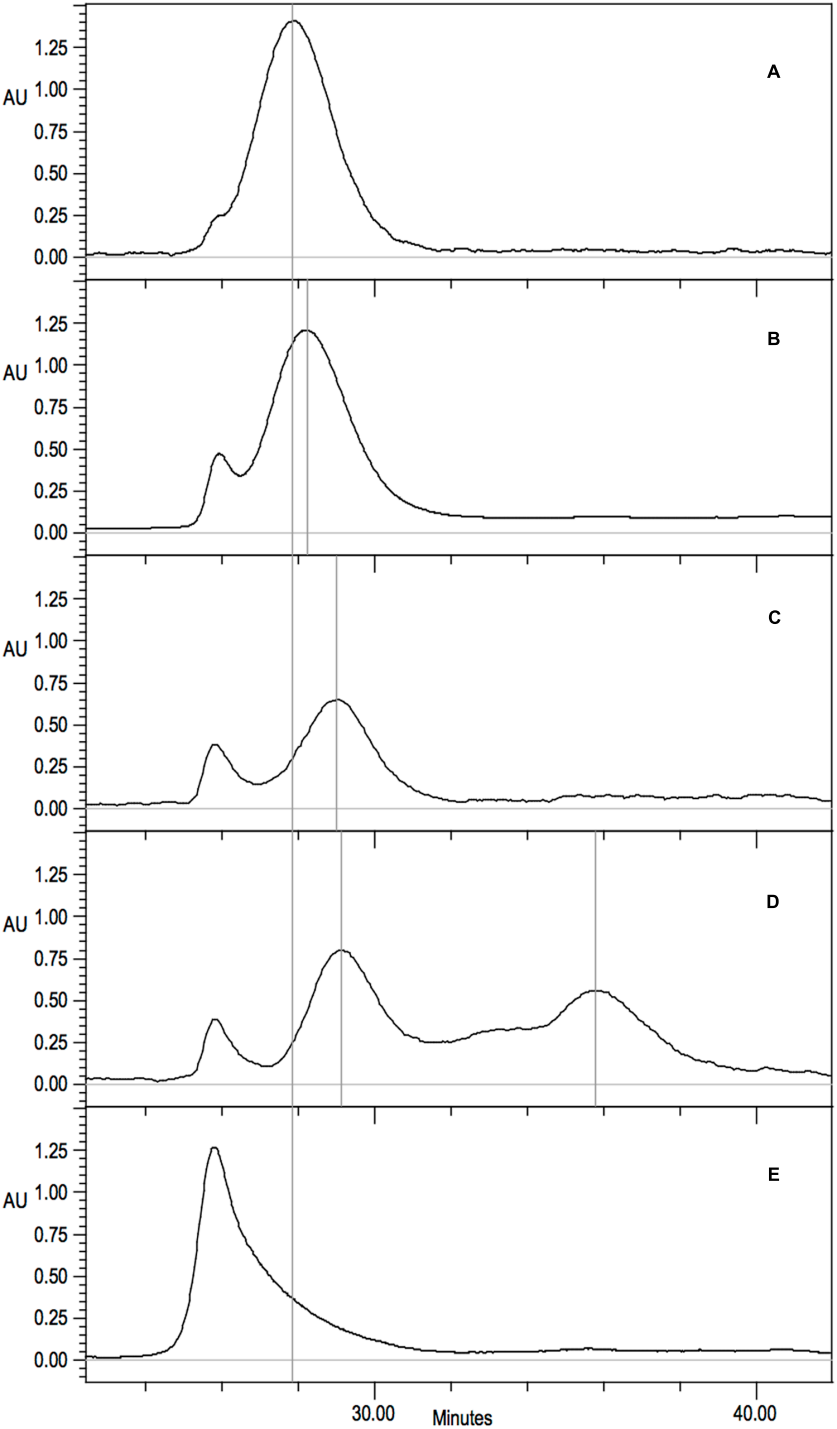
**Gel filtration analysis on Superdex 200 10/300 GL of purified CynD protein in 100 mM MOPS pH 7.7.** Elution monitored as absorbance at 220 nm. Vertical gray lines highlight prominent peaks. Continuous black line indicates wild-type CynD elution peak. Representative elution patterns of substitution mutants in CynD; **(A)** wild type 18-mer CynD, **(B)** intermediate 16-mer mutants (F69C shown) (see **Table 2**), **(C)** *P. stutzeri* CynD 14-mer-like F57C, **(D)** multiple elution peaks of R67C (also see **Figure 6A**), **(E)** sloping void peak of Q228C.

His-tag purified protein from each of the C-surface region mutants was examined by size exclusion chromatography to determine the sizes and distribution of oligomeric variants present. Wild-type CynD from *B. pumilus* (Jandhyala et al., 2003) eluted as a single peak at pH8 and was used to calibrate the elution of the normal CynD 18-mer spiral (**Figure 6A**). CynD from *Pseudomonas stutzeri* (Sewell et al., 2003) eluted as a single peak and was used to calibrate the elution of the 14-mer spiral (**Figure 6C**).

The elution patterns of the substitution mutants fell into a number of different categories (**Table 2**). The majority eluted at the same position as the wild-type *B. pumilus* CynD with a single strong peak consistent with a self-terminating 18-mer (**Figure 6A**). Eleven out of fourteen substitutions in C-surface region 2 had wild type elution patterns. Among the C-surface region 1 mutants only half of the 18 positions tested eluted like the wild type CynD 18mer. Two mutants (F57C, G61C) eluted similar to the *P. stutzeri* enzyme suggesting a failure to extend longer than the *P. stutzeri* CynD 14-mer (**Figure 6C**). Eight mutants eluted intermediate to the wild type CynD 18-mer and the *P. stutzeri* 14-mer (**Figure 6B**). These mutants may represent a 16-mer spiral, though this would imply that the spiral dyad axis does not pass through the A surface, which would differ from the other terminating spiral nitrilases (Wang et al., 2012). One high activity variant (Q228C) eluted primarily in the void volume but with a broad shoulder (**Figure 6E**).

**Table 2:** Elution patterns of each mutant investigated by gel filtration is shown by X.

Peak pattern/mutant	wt	P55C	W56C	F57C	A58C	F59C	I60C	G61C	H62C	P63C	E64C	Y65C	T66C	R67C	K68C	F69C	Y70C	H71C	E72C
18-mer	X	X			X	X	X		X	X		X			X				X
∼16-mer			X								X		X			X	X	X	
14-mer				X				X						X					
Dimer, hexamer, dimer														X					
Extended or aggregated																			

**Peak pattern/mutant**	**wt**	**E222C**	**M223C**	**I224C**	**C225A**	**L226C**	**T227C**	**Q228C**	**E229C**	**Q230C**	**R231C**	**D232C**	**Y233C**	**F234C**	**E235C**	

18-mer	X	X	X	X	X	X	X		X	X	X	X			X	
∼16-mer													X	X		
14-mer																
Multiple smaller																
Extended or aggregated								X								


*Representative chromatographs of each elution pattern shown in **Figure 3.***

**FIGURE 7:**
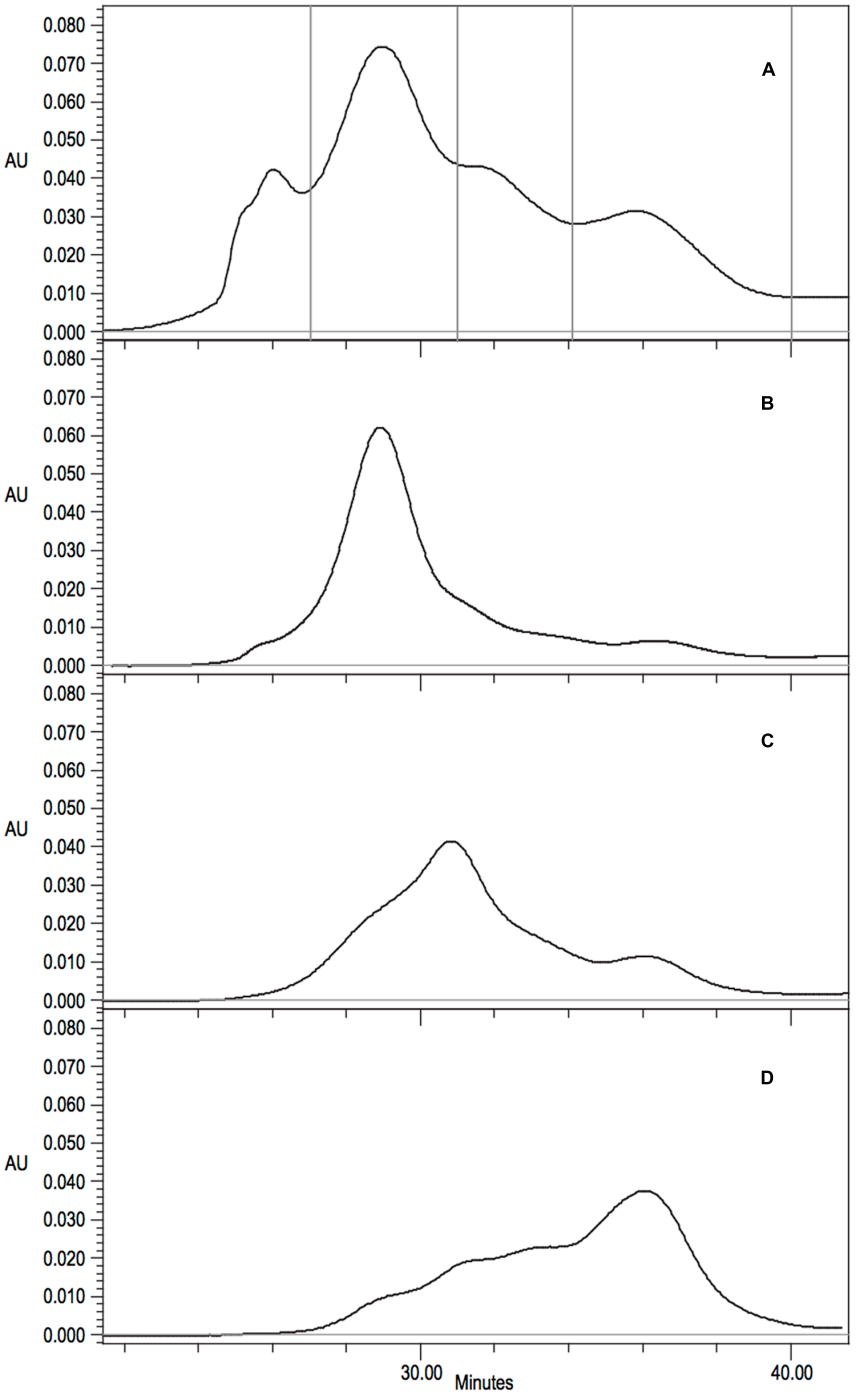
**(A)** Gel filtration analysis and fraction collection on Superdex 200 10/300 GL of CynD R67C protein. Collection periods shown by vertical lines. **(B-D)** Gel filtration of concentrated fractions; **(B)** 14-mer like 27-31 min, **(C)** intermediate decamer-hexamer range 31-34 min, **(D)** dimer 34-40 min.

The mutant R67C eluted as multiple peaks (**Figure 6D**) and the most abundant peak eluted similar to the *P. stutzeri* CynD 14-mer at about 518kDa. Another distinct peak appeared in the range of a dimer (74-111 kDa). There was significant overlap between these two prominent peaks. This intermediate signal appeared as a shoulder in the range of decamer (370 kDa) or a hexamer (222 kDa) (**Figures 6D** and **7A**). R67C was the only C-surface mutation to show a defect in association of the dimer into the larger oligomer.

The elution of CynD R67C as multiple peaks could indicate that the protein was freely assembling and disassembling into varied sizes of oligomers. Alternatively, this elution pattern might represent varied but fixed sizes that are not assembling or disassembling into other sizes at any appreciable rate. To distinguish between these two hypotheses, protein was collected from the different elution peaks of CynD R67C (**Figure 7A**). Fractions were collected and pooled from the 14-mer peak (minute 27-31), the intermediate elution volume (minute 31-34), and from the late peak (minute 34-40). Each fraction was concentrated to 2.00 mg/ml and stored overnight at 4°C to allow any redistribution of the oligomer sizes to occur. Each fraction was then subjected to a second round of gel filtration to see if the fractions had redistributed or remained primarily in the size ranges they were isolated from.

Upon re-chromatography the first fraction remained mostly as the large peak about 518 kDa with a shoulder into the intermediate sized oligomers and some signal through the lower ranges (**Figure 7B**). The intermediate (**Figure 7C**) and the late fraction (**Figure 7D**) separated into multiple peaks but the signal was primarily concentrated within the collected range of the fractions. For all three fractions the majority of the protein retained the original oligomeric size through a second round of gel-filtration. Protein outside the target range in the re-separation is likely due to incomplete resolution of the initial sample. The initial samples and all subsequent fractions of CynD R67C were inactive. These results suggest that the inactive oligomer varieties formed from CynD R67C are stable.

### Analysis for Inter-Subunit Cross-Linking

When analyzed by non-reducing SDS-PAGE gel, all C-surface cysteine scanning mutants yielded only the 40 kDa species, indicating that cross-linking via disulfide bridge formation did not occur (data not shown). A serendipitously discovered mutation at the CynD C-terminus (328EKV to 328SLTTRAPPPPPLRTGC frameshift mutation) with an additional cysteine at the very c-terminus yielded both monomer (40 kDa) and dimer (80 kDa) bands and served as the positive control for this experiment.

### Electron Microscopy

**FIGURE 8:**
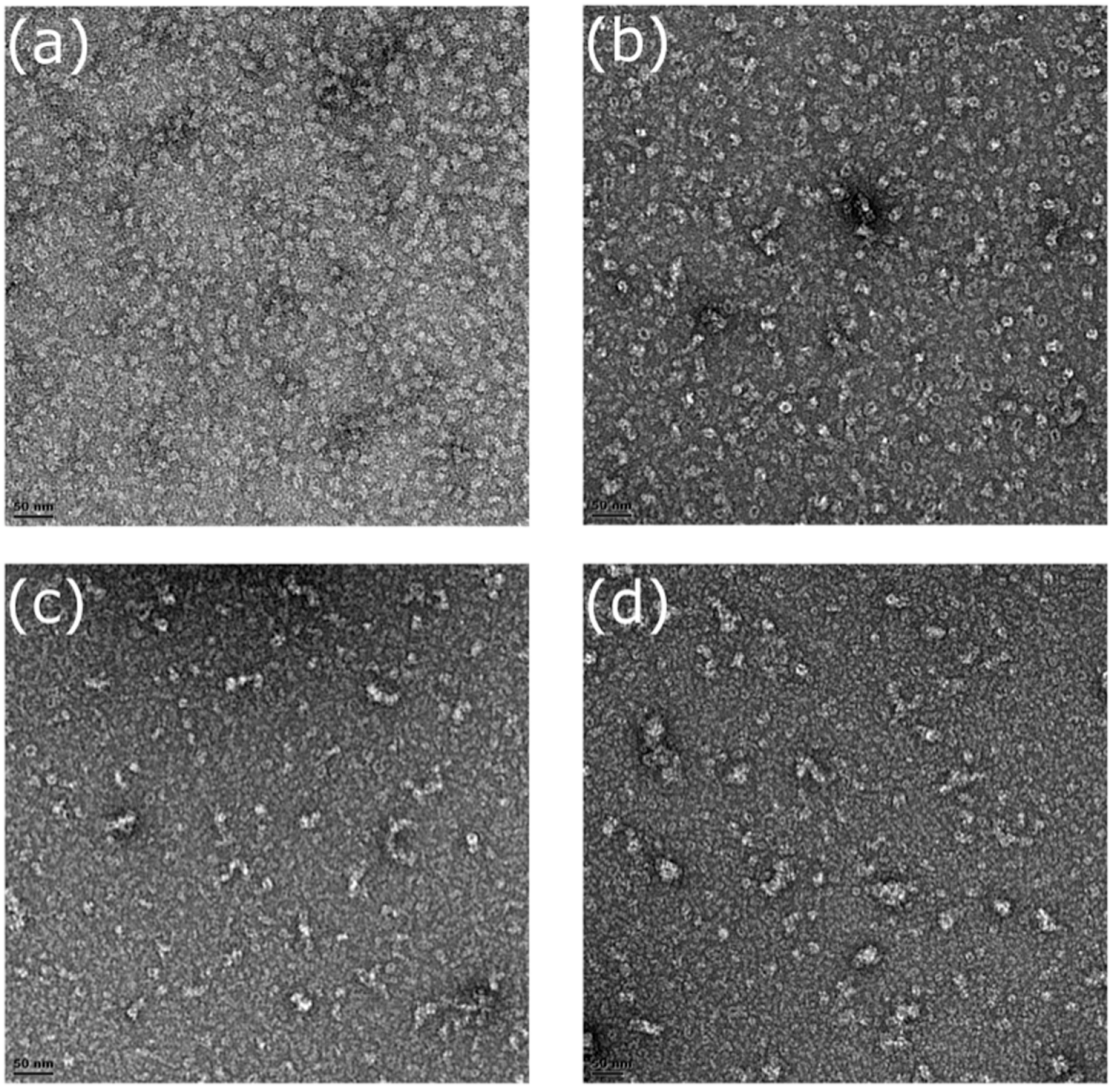
**Fractions of CynD R67C from gel filtration incubated at pH 8.0, stained with 2% uranyl acetate and examined by transmission electron microscopy.** **(A,B)** Represent early fraction and **(C,D)** represent later fractions.

**FIGURE 9:**
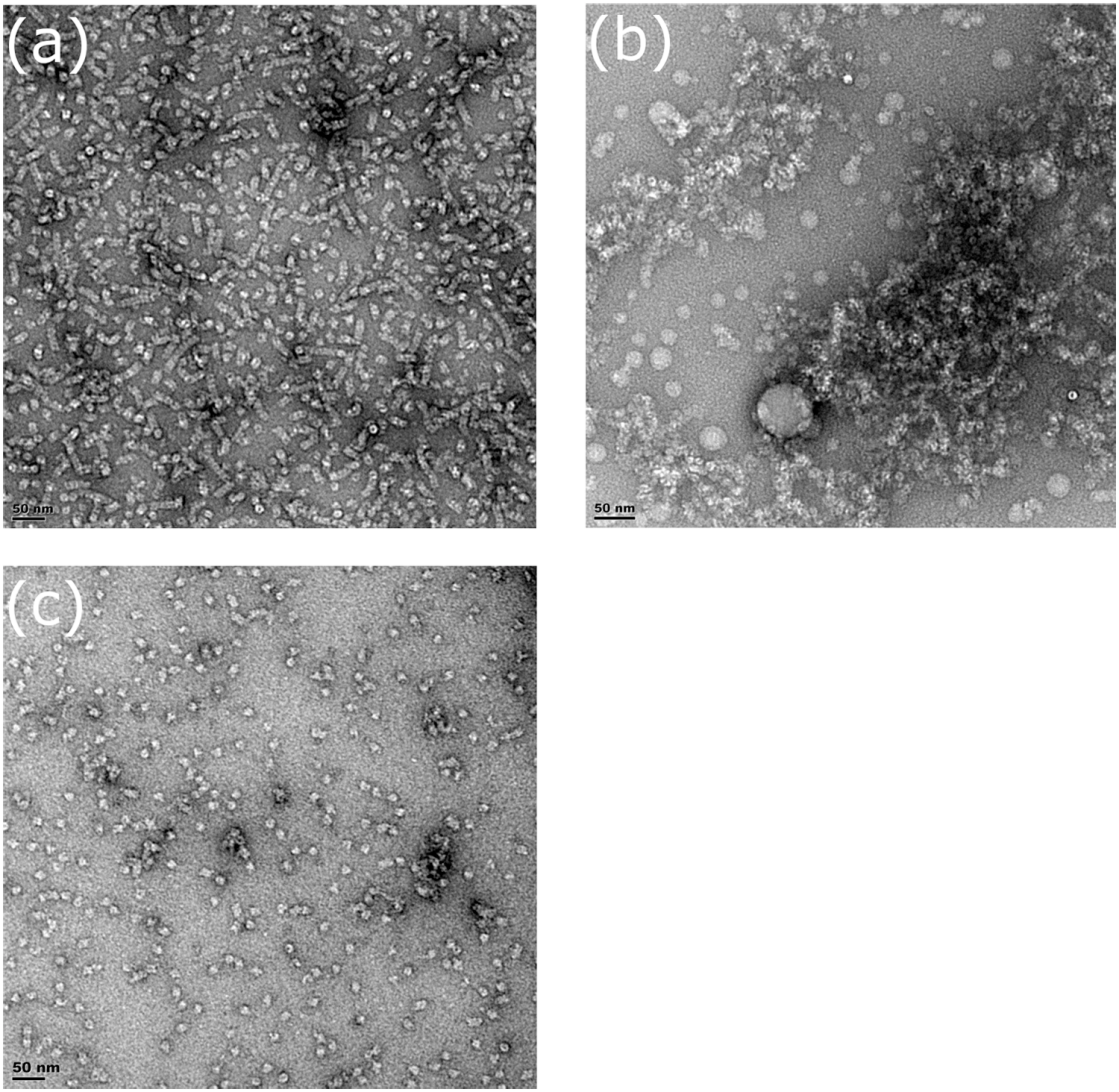
**Fractions of **(A)** CynD Y70C, **(B)** Q228C, and **(C)** wild type from gel filtration incubated at pH 8.0, stained with 2% uranyl acetate and examined by transmission electron microscopy**.

Fractions for three mutants (R67C, Y70C, and Q228C) were collected during gel filtration and examined by transmission electron microscopy (TEM) to confirm oligomer size and to observe their shape. The CynD R67C early fractions (**Figures 8A,B**) showed small terminating spirals and lock-washer rings, while later fractions (**Figures 8C,D**) showed a mixture of C-shaped and smaller oligomers. This is consistent with the multiple peaks seen during size exclusion (**Figure 6D**). The peak fraction of CynD Y70C revealed well formed, self-terminated spiral oligomers (**Figure 9A**), typical of wild type protein (**Figure 9C**). The Q228C variant protein was observed to be aggregates of short spirals and did not form extended helical oligomers (**Figure 9B**).

## Discussion

All crystal structures from the nitrilase-superfamily have a common αββα monomer. With one exception (PDB id: 3ILV, unpublished), the monomers pair to form αββα-αββα dimers (Nakai et al., 2000; Pace et al., 2000; Wang et al., 2001; Kumaran et al., 2003; Kimani et al., 2007; Lundgren et al., 2008). Among members of the nitrilase branch of this superfamily, this basic dimer structure is expanded to form large spiral shaped homo-oligomers (Sewell et al., 2003, 2005; Thuku et al., 2007; Woodward et al., 2008; Dent et al., 2009; Williamson et al., 2010). The interaction between adjacent dimers in these spiral structures occurs at the C-surface and is proposed to be the primary contributor to spiral formation (Sewell et al., 2003). Nitrilases have two insertions relative to the crystal structures of non-spiral-forming homologues (**Figure 2**) that are predicted by modeling to participate in the C-surface interaction (Sewell et al., 2003). One of these insertions (222-235) was visualized in the crystal structure of the C shaped oligomer of β-alanine synthase (βaS) from *Drosophila melanogaster* (Lundgren et al., 2008) and both insertions are visualized in crystal structure of the oxy-nitrilase from *Synechocystis* sp. strain PCC6803 (Zhang et al., 2014). The details of the conformations and the interactions between neighboring monomers across this interface differ in these two crystal structures.

With a view to obtaining insight into the conformation and function of the C surface in CynD, we probed the effect of modifying each of the residues in the two insertion regions to a cysteine. We had initially hoped to introduce a disulfide bridge between the monomers as the residue that was located close to the dyad axis of the interface was mutated. Models based on the fold of βaS suggest that his might occur for L226C and would equally suggest a crosslink between the wild-type Cys225. Alternative models based on the oxy-nitrilase structure suggest that H62C would form the desired crosslink and positively identify the amino acid on the dyad axis. Since neither of these were observed the probability that there are additional structures for the C surface arises and that neither of the observed crystallographic structures of spiral nitrilase superfamily members is a good model for CynD. It is, however, clear that mutation of certain residues in these insertion regions has an effect on activity or oligomerization state or both of these properties.

### Both C-Surface Regions Influence the Oligomeric State of CynD

From cysteine substitutions in the C-surface regions, three types of changes were seen in CynD’s oligomeric state. These included reduction in oligomer size, extension or aggregation, and a partial defect in oligomerization.

Substitutions in both regions revealed mutants that reduced the size of the spiral oligomer from the normal 18-mer of CynD from *B. pumilus* C1. Two mutants (F57C and G61C) eluted with a profile similar to that of the 14-mer of CynD from *P. stutzeri* (Jandhyala et al., 2003; Sewell et al., 2003) whereas other mutants (W56C, E64C, T66C, F59C, Y70C, H71C, Y233C, and F234C) were intermediate between these two. Spiral termination that defines oligomer size in the CynD enzymes is attributed to the alignment and interaction of the E-surface seen in 3D EM reconstructions Sewell et al. (2005). The twist of the spiral determines when these E-surfaces align across the spiral groove. The mutants in the C-surface regions may have changed the spiral length by altering the angle of the dimer-dimer interaction, and thus the pitch of the spiral, leading to interactions at the E-surface which terminate the oligomeric spiral prematurely with different numbers of subunits.

However we can not exclude the possibility that the C-surface mutants have a more direct influence on the terminating interactions. We previously described the mutant Q86R, identified during a screen for activity at high pH that resulted in a loss of self-termination and a dramatic increase in oligomer size. The residue Q86 is thought to participate in the D-surface, which is a cross-spiral interaction that occurs between subunits in adjacent turns of the spiral (Wang et al., 2012). The change in the D-surface could prevent the self-termination by either perturbing or misaligning the E-surfaces.

### The C-Surface is Important in Oligomerization but Probably not in Termination or Size Determination

Apart from changing the size of the oligomer, mutant R67C in C-surface region 1 causes a partial defect in spiral formation. This mutant eluted as a 14-mer followed by possible decamer, hexamer, and dimer peaks (**Figure 6D**). TEM of the different fractions of CynD R67C showed short spirals, ring and C-shaped oligomers, and smaller oligomers (**Figure 8**), consistent with the elution pattern (**Figure 6D**). This pattern indicates that R67C disrupts the oligomerization of dimers and is the only mutation in the current set that produces this effect. Re-chromatography of each size variant peak of R67C indicated that the altered forms were stable and do not re-associate back into the other variant forms (**Figure 7**). The presence of these smaller oligomers may be due to misfolding of the C-surface in a portion of the dimers, resulting in a mixture of prematurely terminated oligomers.

### C-Surface Regions Contain Multiple Positions Critical for CynD Activity

Several mutations at positions within region 1 (55-72) showed at least a 50% reduction in activity (P55, F57, G61, E64, Y65, T66, R67, F69, and Y70; **Figure 5A**). In contrast only two mutated positions (Q230, E235) in region 2 (222-235) reduced activity by ≥ 50%. Kimani et al. (2007) proposed that folding of the C-surface during oligomerization could be activating the nitrilase by positioning a glutamate residue in active site. Changes in the folding of the C-surface in these CynD mutants may put the glutamate out of position sufficiently to slightly perturb activity. In the crystal structure of βaS the C-surface insertion region, when oligomerized, forms the ceiling of the active-site cavity (Lundgren et al., 2008). Interestingly the loop containing the glutamate is disordered in the unmatched dimers at the ends of the βaS oligomer, which could explain the link between activity and quaternary structure in the nitrilase enzymes.

### Many Positions that Altered Oligomerization also Showed Reduced Activity

Of the twelve substitutions that caused changes in oligomerization (**Table 2**), 10 (F57C, G61C, E64C, T66C, R67C, F69C, Y70C, H71C, Y233C, and F234C) had activity at or below 60% of wild type CynD (**Figures 5A,B**), supporting the notion that activation and oligomerization are linked.

Consistent with the relationship between the C-surface and active site cavity in βaS, changes to the twist of the spiral may restrict the active site cavity. From comparisons of plant nitrilases, a correlation was found between spiral twist and substrate size. Woodward suggested that the tight spiral twist of CynD is responsible for its high specificity for cyanide (Woodward, 2011). Five of the mutants in this study with reduced activity (E64C, T66C, R67C, Y70C, and H71C; **Figure 5**) had shortened oligomer sizes as well (**Table 2**).

Two mutants (R67C, and Y70C) had minimal activity within variation of the assay (**Figure 5**). Extended reaction times did not result in further cyanide degradation (data not shown). We cannot rule out some protein retaining partial activity, or substrate binding that could be responsible for some initial cyanide loss in our assay.

While other mutants with reduced oligomer size have reduced activity, CynD Y70C is inactive (**Figure 5A**) yet appears to form normal spiral oligomers as seen by TEM (**Figure 9A**). It is tempting to speculate this residue plays a role in the activation process but it may just as easily change the angle at the C-surface thereby indirectly preventing the activation step.

On the other hand, CynD R67C, which demonstrated a partial defect in oligomerization, is also inactive as purified protein (**Figure 5A**). The activity defect in this mutant is likely directly related to improper oligomerization. While completely inactive as purified protein CynD R67C does show partial activity in cells. The cellular conditions may be allowing CynD R67C to partially oligomerize in an active form, which is disrupted upon lysis, although the protein does not appear to degrade more rapidly than wild type. The R67C defect has also been shown to be suppressed by stabilizing changes at the c-terminus of the protein, supporting the conclusion that only oligomer formation is perturbed (Crum et al., 2015).

## Conclusion

Models of the spiral-forming nitrilase, CynD, based on homology with the crystal structures of two spiral-forming nitrilase superfamily members locate two sequence insertions relative to non spiral-formers in the interface that leads to spiral formation. Mutation at a number of sites in these insertions led to loss of activity or changes in the size of the oligomer or both of these effects. Also located in the interface is a structural element that contains a glutamate residue that is hydrogen bonded to the putative active site lysine. This could explain the effects of the mutations that could change the interface structure on enzyme activity. The fact that activity was more frequently affected by mutation of residues in region 1 suggests that the structure of the interface is more similar to that of the oxy-nitrilase from *Synechocystis* sp. strain Pcc6803 than that of the beta-alanine synthase (βaS) from *Drosophila melanogaster*. However, a complete explanation for the detailed effects of the mutations is beyond the simplistic homology modeling that we have used and must await a high resolution experimental structure determination.

## Author Contributions

JP conducted the majority of experiments, wrote and edited manuscript, prepared most figures. AM conducted all the electron microscopy and prepared those figures and section of the manuscript. TS and MB supervised the research and substantially edited and revised the manuscript.

## Conflict of Interest Statement

The authors declare that the research was conducted in the absence of any commercial or financial relationships that could be construed as a potential conflict of interest.

## References

[B1] Abou Nader M. (2012). *Directed Evolution of Cyanide Degrading Enzymes.* Ph.D. dissertation, Texas A&M University, College Station, TX.

[B2] Akcil A. (2003). Destruction of cyanide in gold mill eﬄuents: biological versus chemical treatments. *Biotechnol. Adv.* 21 501–511. 10.1016/S0734-9750(03)00099-5 14499151

[B3] Crum M. A. Park J. M. Sewell B. T. Benedik M. J. (2015). C-terminal hybrid mutant of *Bacillus pumilus* cyanide dihydratase dramatically enhances thermal stability and pH tolerance by reinforcing oligomerization. *J. Appl. Microbiol.* 118 881–889. 10.1111/jam.12754 25597384

[B4] Dent K. C. Weber B. W. Benedik M. J. Sewell B. T. (2009). The cyanide hydratase from *Neurospora crassa* forms a helix which has a dimeric repeat. *Appl. Microbiol. Biotechnol.* 82 271–278. 10.1007/s00253-008-1735-4 18946669

[B5] Gong J. S. Lu Z. M. Li H. Shi J. S. Zhou Z. M. Xu Z. H. (2012). Nitrilases in nitrile biocatalysis: recent progress and forthcoming research. *Microb. Cell Fact.* 11 142 10.1186/1475-2859-11-142 PMC3537687 23106943

[B6] Goujon M. Mcwilliam H. Li W. Valentin F. Squizzato S. Paern J. (2010). A new bioinformatics analysis tools framework at EMBL,ÄìEBI. *Nucleic Acids Res.* 38 W695–W699. 10.1093/nar/gkq313 20439314 PMC2896090

[B7] Jandhyala D. Berman M. Meyers P. R. Sewell B. T. Willson R. C. Benedik M. J. (2003). CynD, the cyanide dihydratase from *Bacillus pumilus*: gene cloning and structural studies. *Appl. Environ. Microbiol.* 69 4794–4805. 10.1128/AEM.69.8.4794-4805.2003 12902273 PMC169136

[B8] Jandhyala D. M. Willson R. C. Sewell B. T. Benedik M. J. (2005). Comparison of cyanide-degrading nitrilases. *Appl. Microbiol. Biotechnol.* 68 327–335. 10.1007/s00253-005-1903-8 15703908

[B9] Jones D. T. (1999). Protein secondary structure prediction based on position-specific scoring matrices. *J. Mol. Biol.* 292 195–202. 10.1006/jmbi.1999.3091 10493868

[B10] Kimani S. W. Agarkar V. B. Cowan D. A. Sayed M. F. Sewell B. T. (2007). Structure of an aliphatic amidase from *Geobacillus pallidus* RAPc8. *Acta Crystallogr. D Biol. Crystallogr.* 63 1048–1058. 10.1107/S090744490703836X 17881822

[B11] Korte F. Coulston F. (1995). From single-substance evaluation to ecological process concept: the dilemma of processing gold with cyanide. *Ecotoxicol. Environ. Saf.* 32 96–101. 10.1006/eesa.1995.1091 8565884

[B12] Kumaran D. Eswaramoorthy S. Gerchman S. E. Kycia H. Studier F. W. Swaminathan S. (2003). Crystal structure of a putative CN hydrolase from yeast. *Proteins* 52 283–291. 10.1002/prot.10417 12833551

[B13] Larkin M. A. Blackshields G. Brown N. P. Chenna R. Mcgettigan P. A. Mcwilliam H. (2007). Clustal W and Clustal X version 2.0. *Bioinformatics* 23 2947–2948. 10.1093/bioinformatics/btm404 17846036

[B14] Lundgren S. Lohkamp B. Andersen B. Piskur J. Dobritzsch D. (2008). The crystal structure of beta-alanine synthase from *Drosophila melanogaster* reveals a homooctameric helical turn-like assembly. *J. Mol. Biol.* 377 1544–1559. 10.1016/j.jmb.2008.02.011 18336837

[B15] Meyers P. R. Rawlings D. E. Woods D. R. Lindsey G. G. (1993). Isolation and characterization of a cyanide dihydratase from *Bacillus pumilus* C1. *J. Bacteriol.* 175 6105–6112. 8407782 10.1128/jb.175.19.6105-6112.1993 PMC206703

[B16] Nagasawa T. Wieser M. Nakamura T. Iwahara H. Yoshida T. Gekko K. (2000). Nitrilase of *Rhodococcus rhodochrous* J1. Conversion into the active form by subunit association. *Eur. J. Biochem.* 267 138–144. 10.1046/j.1432-1327.2000.00983.x 10601860

[B17] Nakai T. Hasegawa T. Yamashita E. Yamamoto M. Kumasaka T. Ueki T. (2000). Crystal structure of N-carbamyl-D-amino acid amidohydrolase with a novel catalytic framework common to amidohydrolases. *Struct. Fold. Des.* 8 729–737. 10.1016/S0969-2126(00)00160-X 10903946

[B18] Pace H. C. Hodawadekar S. C. Draganescu A. Huang J. Bieganowski P. Pekarsky Y. (2000). Crystal structure of the worm NitFhit Rosetta Stone protein reveals a Nit tetramer binding two Fhit dimers. *Curr. Biol.* 10 907–917. 10.1016/S0960-9822(00)00621-7 10959838

[B19] Pettersen E. F. Goddard T. D. Huang C. C. Couch G. S. Greenblatt D. M. Meng E. C. (2004). UCSF chimera – a visualization system for exploratory research and analysis. *J. Comput. Chem.* 25 1605–1612. 10.1002/jcc.20084 15264254

[B20] Sali A. Blundell T. L. (1993). Comparative protein modeling by satisfaction of spatial restraints. *J. Mol. Biol.* 234 779–815. 10.1006/jmbi.1993.1626 8254673

[B21] Schreiner U. Hecher B. Obrowsky S. Waich K. Klempier N. Steinkellner G. (2010). Directed evolution of *Alcaligenes faecalis* nitrilase. *Enzyme Microb. Technol.* 47 140–146. 10.1016/j.enzmictec.2010.05.012

[B22] Sewell B. T. Berman M. N. Meyers P. R. Jandhyala D. Benedik M. J. (2003). The cyanide degrading nitrilase from *Pseudomonas stutzeri* AK61 Is a two-fold symmetric, 14-subunit spiral. *Structure* 11 1413–1422. 10.1016/j.str.2003.10.005 14604531

[B23] Sewell B. T. Thuku R. N. Zhang X. Benedik M. J. (2005). Oligomeric structure of nitrilases: effect of mutating interfacial residues on activity. *Ann. N. Y. Acad. Sci.* 1056 153–159. 10.1196/annals.1352.025 16387684

[B24] Singh R. Sharma R. Tewari N. Rawat D. S. (2006). Nitrilase and its application as a ‘green’ catalyst. *Chem. Biodivers.* 3 1279–1287. 10.1002/cbdv.200690131 17193242

[B25] Thuku R. N. Brady D. Benedik M. J. Sewell B. T. (2009). Microbial nitrilases: versatile, spiral forming, industrial enzymes. *J. Appl. Microbiol.* 106 703–727. 10.1111/j.1365-2672.2008.03941.x 19040702

[B26] Thuku R. N. Weber B. W. Varsani A. Sewell B. T. (2007). Post-translational cleavage of recombinantly expressed nitrilase from *Rhodococcus rhodochrous* J1 yields a stable, active helical form. *FEBS J.* 274 2099–2108. 10.1111/j.1742-4658.2007.05752.x 17371547

[B27] Wang L. Watermeyer J. M. Mulelu A. E. Sewell B. T. Benedik M. J. (2012). Engineering pH-tolerant mutants of a cyanide dihydratase. *Appl. Microbiol. Biotechnol.* 94 131–140. 10.1007/s00253-011-3620-9 21993481

[B28] Wang W. C. Hsu W. H. Chien F. T. Chen C. Y. (2001). Crystal structure and site-directed mutagenesis studies of N-carbamoyl-D-amino-acid amidohydrolase from *Agrobacterium radiobacter* reveals a homotetramer and insight into a catalytic cleft. *J. Mol. Biol.* 306 251–261. 10.1006/jmbi.2000.4380 11237598

[B29] Watanabe A. Yano K. Ikebukuro K. Karube I. (1998). Cyanide hydrolysis in a cyanide-degrading bacterium, *Pseudomonas stutzeri* AK61, by cyanidase. *Microbiology* 144(Pt 6), 1677–1682. 10.1099/00221287-144-6-1677 9639937

[B30] Waterhouse A. M. Procter J. B. Martin D. M. A. Clamp M. Barton G. J. (2009). Jalview Version 2-a multiple sequence alignment editor and analysis workbench. *Bioinformatics* 25 1189–1191. 10.1093/bioinformatics/btp033 19151095 PMC2672624

[B31] Williamson D. S. Dent K. C. Weber B. W. Varsani A. Frederick J. Thuku R. N. (2010). Structural and biochemical characterization of a nitrilase from the thermophilic bacterium, *Geobacillus pallidus* RAPc8. *Appl. Microbiol. Biotechnol.* 88 143–153. 10.1007/s00253-010-2734-9 20607233

[B32] Woodward J. D. (2011). *The Relationship between Structure and Specificity in the Plant Nitrilases.* Ph.D. dissertation, University of Cape Town, Cape Town.

[B33] Woodward J. D. Weber B. W. Scheffer M. P. Benedik M. J. Hoenger A. Sewell B. T. (2008). Helical structure of unidirectionally shadowed metal replicas of cyanide hydratase from *Gloeocercospora sorghi*. *J. Struct. Biol.* 161 111–119. 10.1016/j.jsb.2007.09.019 17997328

[B34] Zhang L. J. Yin B. Wang C. Jiang S. Q. Wang H. L. Yuan Y. A. (2014). Structural insights into enzymatic activity and substrate specificity determination by a single amino acid in nitrilase from *Synechocystis* sp PCC6803. *J. Struct. Biol.* 188 93–101. 10.1016/j.jsb.2014.10.003 25450592

